# Fully Polymeric Domes as High-Stroke Biasing System for Soft Dielectric Elastomer Actuators

**DOI:** 10.3389/frobt.2021.695918

**Published:** 2021-06-10

**Authors:** Julian Neu, Jonas Hubertus, Sipontina Croce, Günter Schultes, Stefan Seelecke, Gianluca Rizzello

**Affiliations:** ^1^ Department of Systems Engineering, Saarland University, Saarbrücken, Germany; ^2^ Department of Material Science, Saarland University, Saarbrücken, Germany; ^3^ Department of Sensors and Thin Films, University of Applied Sciences of Saarland, Saarbrücken, Germany

**Keywords:** dielectric elastomer, dielectric elastomer actuator, soft actuator, polymeric domes, bi-stable biasing mechanism, flexible biasing system

## Abstract

The availability of compliant actuators is essential for the development of soft robotic systems. Dielectric elastomers (DEs) represent a class of smart actuators which has gained a significant popularity in soft robotics, due to their unique mix of large deformation (>100%), lightweight, fast response, and low cost. A DE consists of a thin elastomer membrane coated with flexible electrodes on both sides. When a high voltage is applied to the electrodes, the membrane undergoes a controllable mechanical deformation. In order to produce a significant actuation stroke, a DE membrane must be coupled with a mechanical biasing system. Commonly used spring-like bias elements, however, are generally made of rigid materials such as steel, and thus they do not meet the compliance requirements of soft robotic applications. To overcome this issue, in this paper we propose a novel type of compliant mechanism as biasing elements for DE actuators, namely a three-dimensional polymeric dome. When properly designed, such types of mechanisms exhibit a region of negative stiffness in their force-displacement behavior. This feature, in combination with the intrinsic softness of the polymeric material, ensures large actuation strokes as well as compliance compatibility with soft robots. After presenting the novel biasing concept, the overall soft actuator design, manufacturing, and assembly are discussed. Finally, experimental characterization is conducted, and the suitability for soft robotic applications is assessed.

## 1 Introduction

Dielectric Elastomers (DEs) are a type of electro-mechanical transducers which react to an electric voltage with a significant change in shape. A typical DE consists of a thin polymeric membrane, whose thickness ranges from few tens to a few hundreds of micrometers, coated with compliant electrodes on both sides. Commonly used materials for the elastomer are acrylics and silicone (Chiba, 2014), while the electrodes can be fabricated with a mixture of carbon powder and silicone oil (Kornbluh et al., 2004) as well as stretchable metallic thin films (Hubertus et al., 2020). When a high voltage on the order of several kV is applied to the electrodes, charges of opposite signs are stored onto them. These charges induce electrostatic forces which let the electrodes attract each other. Such an effect leads to a thinning of the membrane which, in turn, is followed up by an area expansion, as a result of the incompressibility of the silicone (Pelrine et al., 2001b).

The principle described above makes DEs a promising technology for electrically-controllable actuators. Features such as large strain (over 100%), as well as high energy efficiency and density (Kornbluh et al., 2004), enabled the development of DE-based mechatronic devices which are capable of performance not achievable with competing technologies (Pelrine et al., 2001a; York et al., 2013). The possibility of structuring the compliant electrodes in various ways, together with the material overall flexibility, has led researchers to develop a wide spectrum of DE actuator (DEA) solutions. Some authors demonstrated how DEAs can be used to manufacture lightweight fluid pumps (Mohd Ghazali et al., 2017; Cao et al., 2019; Linnebach et al., 2020), or showcased the possibility of generating high forces (Hau et al., 2018b). Further examples of DEA prototypes range from DE driven loudspeakers (Rustighi et al., 2018), contactors (Linnebach et al., 2019), and valves (Hill et al., 2017) to jumping devices (Luo et al., 2020) and medical systems (Goulbourne et al., 2003), to mention a few.

Due to their intrinsically high compliance, DEs have also been extensively used as actuators for soft robots. Soft robotics is a current research topic that deals with the investigation and development of highly flexible robots, by emulating bio-inspired actuation concepts (Albu-Schaffer et al., 2008). The high flexibility and compliance which characterize soft robots ensure a safer interaction with humans, as well as the possibility to achieve complex deformation patterns. Examples in this field range from bending arm-like structures (Xing et al., 2020) to crawling or rolling DE based-robots (Duduta et al., 2017; Henke et al., 2017; Li et al., 2018), as well as swimming jellyfish robots (Godaba et al., 2016). It can be noted, however, that many of the above mentioned actuators exploit a locomotion mode to generate a sufficient movement of the device. Despite being suitable for certain target applications or goals, locomotion does not fully exploit the high strain potential of the material.

To properly design soft DEA solutions which enable full exploitation of the material large stroke capability, more advanced design solutions must be considered. Hodgins et al. (2011) showed that, by coupling a DE membrane with an appropriate mechanical biasing system (e.g., a mass, a spring), the resulting stroke of the actuator can be remarkably increased. This concept has been extensively exploited in conventional DEA systems (Wang et al., 2009; Berselli et al., 2010; Lu et al., 2016), as well as in some DEA-driven soft robots (Zhang et al., 2006; Kofod et al., 2007). A further comparative study conducted in Hodgins et al. (2013) revealed that, if the DE biasing mechanisms exhibits a region with negative slope (i.e., stiffness) within its force-displacement characteristics, it allows to achieve a drastically higher stroke and work output compared to conventional mass- and linear spring-type bias mechanisms. Such negative-rate biasing spring (NBS) element can be practically manufactured in many ways, e.g., via a pre-compressed steel buckled beam (Hodgins and Seelecke, 2010), attracting permanent magnets (Loew et al., 2018), or more advanced types of mechanisms (Berselli et al., 2011). NBS elements have been extensively used to improve the performance of simple DEA configurations, e.g., single degree-of-freedom industrial actuators (Berselli et al., 2011; Hau et al., 2018b; Linnebach et al., 2019). The biasing systems used in most of the current DE soft robots, however, lack the above mentioned NBS principle, thus they do not allow to exploit the high flexibility of the DE to generate soft actuators with large stroke. This is possibly due to the rigid materials commonly used to manufacture the NBS itself, which are mostly based on metal, as well as due to the stiff frames and housings required to implement mechanical clamping and connections. Both, metal based-NBS elements and rigid clamping components represent then additional stiff parts, whose characteristics are not compatible with the compliance requirements of soft robotics. Due to these reasons, the development of large-stroke soft DEA components, which effectively integrate the benefits of NBS elements while keeping at the same time an overall compliant structure, is still an unexplored field.

To overcome the above mentioned issue, and thus enable large-stroke DE soft actuators for robotic and wearable applications, a compliant and flexible NBS element is needed, which does not require using additional rigid components. In this paper, we propose a novel type of NBS biasing element for DEAs, based on three-dimensional domes manufactured out of soft polymeric material (Young’s modulus on the order of a few tens of kPa). Earlier research works have already demonstrated that, by properly designing the geometry of such domes, a region of negative stiffness can be induced in their force-displacement characteristics (Madhukar et al., 2014; Alturki and Burgueño, 2019). In this way, a soft and fully polymer-based NBS is achieved. Due to the compliance matching between DE and dome materials, this solution permits in principle to achieve a fully soft and large-stroke actuator system. Those types of mechanisms, however, have not been previously adopted as biasing elements for DE membranes, to the best of our knowledge. The present manuscript represents then the first systematic investigation of soft biasing system concepts for high-stroke DEAs, which leads to potential applications in soft robotics as well as wearables.

After presenting the dome concept, its design and manufacturing process will be discussed. Based on experimental characterization of both DE and biasing element, a heuristic approach is then proposed for finding the dome design which maximizes the overall DEA stroke. The optimized DEA is then assembled and characterized experimentally via an extensive set of experiments, aimed at evaluating its static and dynamic performance, as well as its ability to operate while being stretched and deformed. The ability of the dome-DEA system to perform a stroke under a deformation/stretching state opens up the perspective to manufacture completely flexible micro-arrays of DEAs. This novel technology could be potentially used to develop soft and flexible smart skins for soft robots, as well as wearable surfaces, that exhibit not only cooperative actuation capabilities but can also sense contact forces and distributed displacements. Despite flexible DEA arrays have already been presented in recent research (Marette et al., 2017), the lack of a soft NBS-type biasing system does not allow to truly exploit the high-stroke potential of the DE material.

We also point out that this paper is an extended version of the work previously presented in Neu et al. (2020). In our previous research, a first DEA prototype based on a silicone dome was presented. In here, we extend the results in Neu et al. (2020) by including:

• a detailed description of the dome manufacturing process;
• a systematic approach to optimize the design of the dome geometry for a specific target application;
• an improved actuator design solution which ensures safer operations regarding the high-voltage;
• an extensive experimental campaign aimed at characterizing the novel dome-DEA system in terms of stroke, dynamic performance, and energy consumption;
• an investigation of the actuator performance while operating in a deformed state.

The structure of the article is organized as follows. After explaining the operating principle of a DEA in Section2, the novel dome-based biasing system is described in Section 3. An optimization procedure for the dome design is presented in Section 4. Manufacturing and performance characterization of the full actuator system are then shown in Section 5. Finally, concluding remarks are discussed in Section 6.

## 2 Dielectric Elastomer Actuators

In this section, the operating principle of DE membranes will be presented first. Then, the role of the biasing system in determining the performance of a DEA is discussed in details.

### 2.1 Basics of Dielectric Elastomers

**FIGURE 1:**
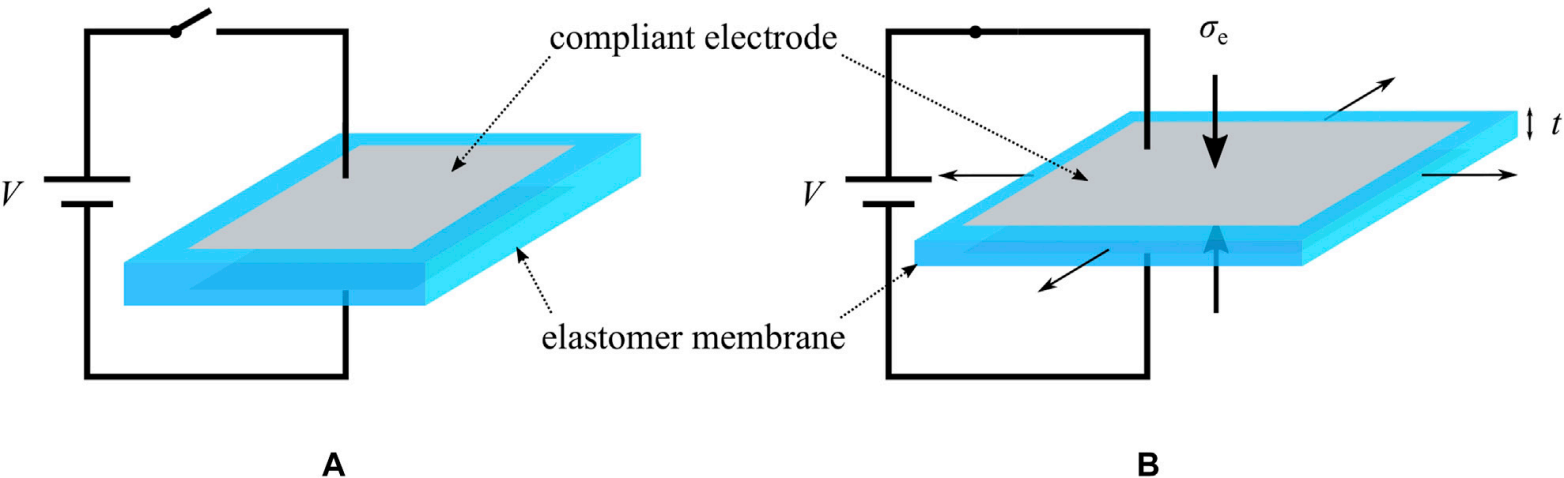
Basic principle of a dielectric elastomer. **(A)** The membrane is initially undeformed. **(B)** When a high voltage is applied to the compliant electrodes, the silicone membrane gets thinner and expands in area.

As described in Section 1, a typical DE consists of a polymer film coated with compliant electrodes on both sides. When a voltage is applied to such a DE, charges of opposite signs are stored in the electrodes. These charges lead to attractive electrostatic forces which cause a thinning of the dielectric film and, due to the incompressibility of the elastomer, an expansion of the membrane area, as shown in Figure 1. The electrically-induced stress on the silicone film $\sigma_{e}$ can be expressed by

$$\sigma_{e}=-\epsilon_{r}\epsilon_{0}{\left(\frac{V}{t}\right)}^{2},$$ (1)

with $\epsilon_{r}$ and $\epsilon_{0}$ representing the relative and vacuum permittivity, respectively, while *V* and *t* are the voltage and the membrane thickness (Pelrine et al., 1998). The minus sign in Eq. 1 denotes the fact that $\sigma_{e}$ always acts as a compressive stress, as also shown in Figure 1B. For further information about the basics of DEs, the reader may refer to Carpi et al. (2008, 2015).

While commercially available materials are generally used for the polymeric film [e.g,. 3M VHB 4910 acrylics as in Frediani et al. (2014), or Wacker Elastosil silicone as in Matysek et al. (2008)], the electrodes can be manufactured in several possible ways (Rosset and Shea, 2013). For instance, it is possible to apply a mixture of silicone oil and carbon black by hand (Lu et al., 2016) or, to achieve a more uniform electrode layer, via screen-printing (Fasolt et al., 2017). A rather new approach, which has been proposed in Hubertus et al. (2020), is based on sputtering a metallic thin film (thickness of 10 nm) onto a pre-stretched silicone membrane. After releasing the pre-stretch, the metallic film forms wrinkles without delamination, and can be deformed even above the level of pre-stretch without losing conductivity. This new type of metallic DE electrode is used in the prototype presented in this manuscript, to showcase its suitability for soft DEAs. Even though the use of metallic electrodes may appear contradictory for the scope of soft robotics, the 10 nm thick sputtered metallic thin film has practically no influence on the stiffness of the DE. In addition, according to Hubertus et al. (2020), the metal electrodes exhibit a sheet resistance which is about two orders of magnitude smaller than the one of usual carbon black electrodes, thus allowing to improve the actuation speed as well as the energy efficiency.

### 2.2 Basics of Dielectric Elastomer Actuators

**FIGURE 2:**
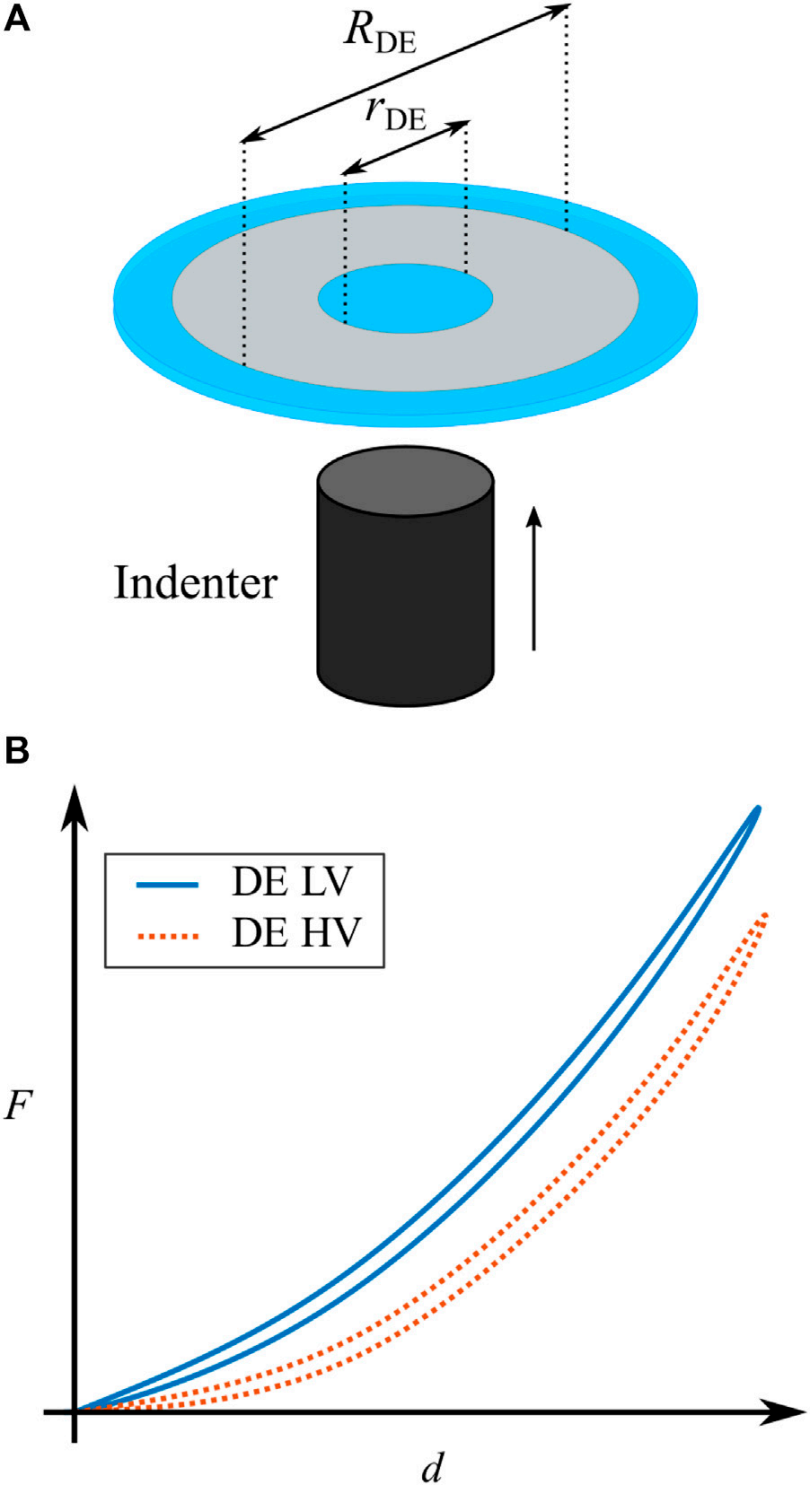
**(A)** Schematic depiction of a COP-DEA, active electrode-covered material (depicted in gray) and passive elastomer material (depicted in blue). **(B)** Qualitative sketch of the two characterizing curves of a COP-DEA. The blue continuous line represents the low voltage curve and the dotted red line represents the high voltage curve.

Due to the high flexibility of DE material, the actuation principle described above can be adapted to several geometries and configurations. In this work, we focus on a specific class of DEA systems, namely a circular out-of-plane DEA (COP-DEA). An example of COP-DEA layout is shown in Figure 2A. The grey part corresponds to the compliant electrodes, while the blue elements represent the passive membrane. The geometry of a COP-DEA is generally defined by the outer and inner radii, denoted as $R_{\text{DE}}$ and $r_{\text{DE}}$, respectively.

For characterizing a COP-DEA, the center passive part (i.e., the inner blue circle in Figure 2A) has to be mechanically deflected out-of-plane, while its reaction force is measured at the same time, as shown in Figure 2A. We denote as *d* and *F* the corresponding out-of-plane displacement and reaction force, respectively. This can be done with and without a voltage applied to the electrodes, thus leading to a pair of force-displacement curves describing the operating range of the actuator. A qualitative example of such characterization curves is reported in Figure 2B. In here, the continuous blue line represents the DE with no or a very low voltage (LV) applied, while the dotted red line describes the case with applied high voltage (HV). The curves basically show that the thinning of the membrane due to the HV-induced activation leads to a softening of the DE. Note also that the curves exhibit a moderate hysteresis under cyclic operations, which is due to the viscoelastic behavior of the elastomer.

In order to exploit the softening effect in Figure 2B for actuation purposes, a mechanical preload must be applied to the membrane. In case of the COP-DEA, such a preload can be obtained by connecting a passive spring element to the center passive part of the membrane. Hodgins et al. (2013) compared different types of biasing elements in terms of resulting maximum stroke. Based on such analysis, it is shown that a NBS has the highest potential for the generation of high strokes, compared to more conventional masses and linear springs.

**FIGURE 3:**
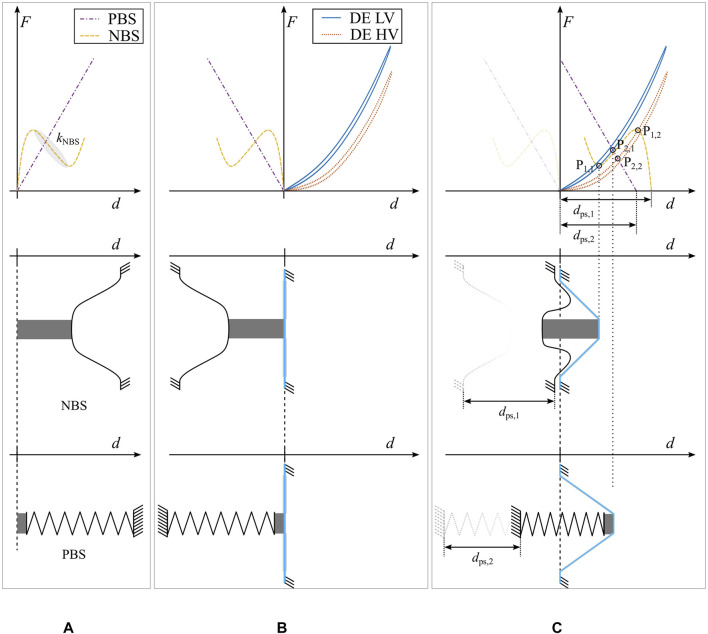
Schematic depiction of the biased DEA. **(A)** shows the force-displacement curves of a PBS and a NBS in the first row. In the lower part, a sketch of the two considered biasing elements is depicted. **(B)** shows the resulting system curves and a sketch of the assembly, when a COP-DEA is brought into contact with the biasing elements. **(C)** represents the system force-displacement curves in the biased state, and shows the resulting configuration in each one of the two sketched assemblies.

To clarify why a NBS outperforms other types of preloading mechanisms, the relationship between biasing mechanism and DEA performance must be properly understood. To this end, it is convenient to study the force-displacement characteristics of the different biasing elements in combination with the curves of the DE, namely a positive-rate biasing spring (PBS) and an example of NBS, both reported in Figure 3. In particular, Figure 3A shows the force-displacement curves of both biasing systems, together with their schematic depiction. The dashed yellow line describes the NBS. In here, the region confined between the two local extrema exhibits a negative slope, which can be characterized by a stiffness parameter $k_{\text{NBS}}$. The dash-dot purple curve represents the PBS, and is solely characterized by its positive slope. In here, a positive displacement denotes the direction of pushing on top of each element, as implied by the arrows.

For assembling a COP-DEA, the biasing element has to be connected to the center passive part of the elastomer membrane. As soon as a contact is established between both parts, with no forces exchanged among them, the resulting system configuration can be described by the diagram in Figure 3B. In here, the grey part represents a spacer, which serves as mechanical connection element. In this assembly, the DE and the biasing elements are working against each other: a deflection of the DE center part in the direction of positive *d* values corresponds to a pulling on the biasing springs, which is why the spring curves are mirrored with respect to the *y*-axis. Note how the application of a HV in this state would not result in a movement of the center part, since no force is being exchanged between DE and biasing element.

In order to generate a stroke the DE membrane and the bias element have to be moved relatively closer to each other, by an amount of $d_{\text{ps},1}$ for the NBS and by $d_{\text{ps},2}$ for the PBS, respectively, as shown in Figure 3C. In the force-displacement diagrams, this corresponds to a shift of the spring curves by the same values $d_{\text{ps},1}$ and $d_{\text{ps},2}$, respectively, as shown in the first row of Figure 3C. In a real-life system, this shift causes the bias element to be compressed, whereas the center part of the DE is deflected out-of-plane. Such an amount of deflection corresponds to the *d* value of points ${\text{P}}_{1,1}$ and ${\text{P}}_{2,1}$, i.e., the intersections between the DE LV curve and the corresponding biasing element. From classical mechanics, it is known that the intersection point between the two force-displacement curves is equivalent to a minimum energy state of the system, which, in turn, represents a stable equilibrium configuration. When the DE is subjected to a HV it becomes softer, and its characteristics changes into the red curve accordingly. When switching from LV to HV, the system equilibrium point will then evolve to the current lowest potential energy state, which is again represented by the intersections between DE and biasing elements curves, i.e., ${\text{P}}_{1,2}$ and ${\text{P}}_{2,2}$.

By comparing the two biasing solutions in Figure 3C, it is now evident that the intersection points of the NBS curve exhibit a much wider separation than the ones of the PBS case. Since the distance between the intersections coincides with the actuator stroke, it can be concluded that a NBS leads to a better performing DEA, compared to a PBS. Note how the key aspect of the NBS is represented by the region of inverted slope $k_{\text{NBS}}$, which needs to be matched with the gap between the two DE curves. Therefore, to properly optimize the design of a NBS, the most important parameters are represented by the slope $k_{\text{NBS}}$ as well as the amount of pre-stretch $d_{\text{ps}}$.

## 3 Novel Dome-Based Biasing System

This section presents the novel biasing solution based on the polymeric dome. After discussing the main idea, a new dome design layout is presented and experimentally characterized.

### 3.1 Basic Concept

**FIGURE 4:**
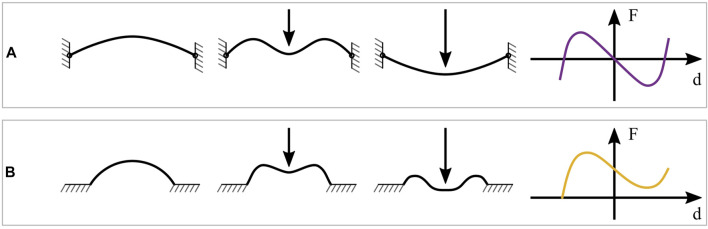
Different realizations of bi-stable mechanisms. **(A)** pre-compressed buckled beam, snapping behavior under a load and corresponding force-displacement curve. **(B)** polymeric dome, snapping behavior under a load and corresponding force-displacement curve.

In DE applications, conventional NBS elements usually consist of thin, pre-compressed metal beams (Hau et al., 2018a). By compressing a beam with an initial length $L_{0}$ to a new length $L<L_{0}$, and clamping its end-points, it will deform into a buckling shape according to the theory of Euler buckling. We now assume that a force is applied to the center part of the beam, directed orthogonally to the beam axis. If the magnitude of such a force is progressively increased, at some point a snap-through will occur, causing the beam to buckle in the opposite direction, as shown in Figure 4A. This phenomenon reflects the bistability due to the beam buckling (Vangbo, 1998). Such a snap-through behavior can be explained by considering the structural potential energy stored in the beam. This energy consists of two main contributions, namely bending energy and compression energy. When the deflection begins, the external force increases both bending and compression energies of the beam. After a certain amount of deflection has occurred, the compression energy reaches its maximum, while the bending energy keeps increasing monotonically. Depending on the beam design, the decrease of compression energy can be faster than the increase of bending one, which results in a negative force and, hence, in a snap-through (Qiu et al., 2004).

Buckled beams which follow this behavior usually exhibit a point-symmetrical force-displacement curve with respect to the unstable equilibrium point, i.e., (d,F)=(0,0). A qualitative example of such a curve is also shown on the right-hand side of Figure 4A. By considering the DEA principle explained in Section 2.2, it becomes evident that such an ideal NBS curve is not optimal to be used as biasing element for DEs. To replicate the ideal shape shown in Figure 3, the NBS curve in Figure 4A must be shifted upwards, so that only one interesction occurs with the horizontal line of F=0 N. Physically, this means that the reaction force pushing on the center is always positive (i.e., tensile), hence it always acts in the opposite direction of the deflection. This desired behavior is favored by using as-fabricated beam designs, which are not forced in a buckling mode by compression, and are thus free of intrinsic stresses (Qiu et al., 2004). Alternatively, a similar effect can be obtained by connecting in parallel an ideal beam with a PBS (Hau et al., 2018b).

The idea of using polymeric domes as negative-stiffness mechanisms, instead of buckled beams, is based on results presented in earlier research works (Madhukar et al., 2014; Alturki and Burgueño, 2019). The use of cast silicone domes allows to combine the stress-free manufacturing together with the softness and flexibility that are required by the biasing system. An example of such a dome, together with its corresponding force-displacement curve, is shown in Figure 4B. In contrast to the force-displacement curve of the buckled beam, the dome characteristic does not show an unstable equilibrium position in the center of the negative branch. Since the curve does not lie below the horizontal line corresponding to F=0 N, the dome reaction force is always acting in the opposite direction to the displacement. This is due to the as-fabricated design. Thus, when deformed, the dome always strives to restore its unique equilibrium state without requiring an additional PBS element. As a result, we conclude that the shape of the dome force-displacement curve makes it a potentially suitable biasing element for COP-DEAs.

### 3.2 Dome Layout

**FIGURE 5:**
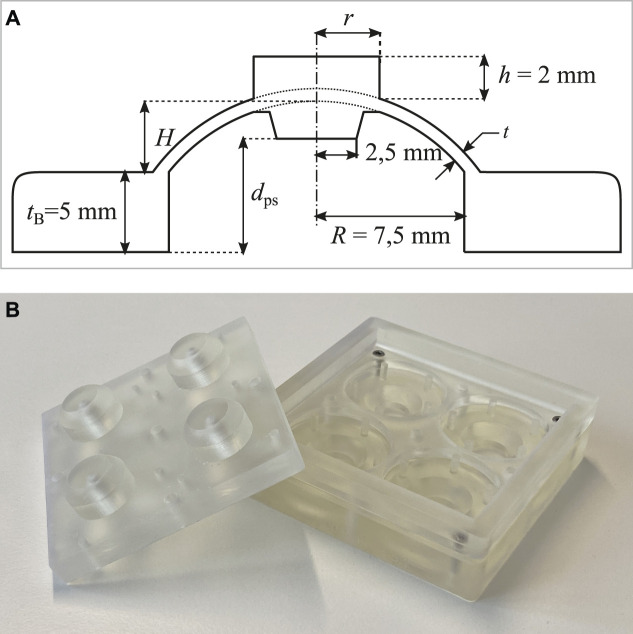
**(A)** Cross-sectional sketch of the dome geometry, with all the relevant parameters. Note how *r*, *t*, and *H* are left free to design. **(B)** Picture of a partly assembled 3D printed mold.

To develop a dome-based NBS concept which is suitable for DE applications, the basic layout presented in Madhukar et al. (2014) is considered as a starting design point. To make such a geometry compatible with the COP-DEA described in Section 2.2, the design in Madhukar et al. (2014) is properly modified by surrounding the isolated dome with a relatively thick base, as well as by adding a round flat-top. A cross-sectional sketch of the modified dome design is reported in Figure 5A. The base serves as a pseudo-solid frame for the dome, while the flat-top is used for shaping its force-displacement characteristic curve. More specifically, increasing the flat-top radius *r* leads to a shorter length of the thin walled region, which results in a higher compression stress given the same deflection of the center part. A larger compression stress is expected to increase the maximum force in the force-displacement curve. Other relevant geometric parameters are the wall thickness *t*, the height of the dome *H*, and the base radius *R*. In order to match the diameter of the sputtered DE, given by $2R_{\text{DE}}=20\;\text{mm}$, the base radius is fixed to the value R=7.5 mm. This choice leaves three free parameters for optimizing the design of the dome geometry, i.e., r,t,and H. The pre-stretch $d_{\text{ps}}$ can be further tuned by adding an extension to the bottom part of the flat-top. As the DE is supposed to be attached to the bottom of the dome base, the value of $d_{\text{ps}}$ has to be referred to the base. The radius of that extension is designed to match the inner radius of the DE, which is given by $r_{\text{DE}}=2.5\;\text{mm}$.

### 3.3 Dome Manufacturing

For manufacturing the domes, a casting process based on a pourable addition-curing silicone rubber that vulcanizes at room temperature [SilGel® 612 EH, mixing ratio 1.5:1 (A:B)] is used. The casting molds consist of three solid parts, which are created with a 3D SLA printer. These parts are printed with a minimum layer height of 25 μm, which also coincides with the value of the printer XY resolution, as specified by the manufacturer. Figure 5B shows a picture of a partly assembled mold. For achieving more comparable results with one casting process, one mold can be used to manufacture four individual dome geometries, which can either be identical to account for manufacturing tolerances, or can possibly differ in one or more parameters.

The manufacturing process consists of the following steps:

1. The silicone is catalyzed with a mixing ratio of 1.5:1 (A:B);
2. The catalyzed silicone is cast into the bottom part of the mold;
3. The mixture is degassed in a vacuum chamber for 5 min;
4. The top part of the mold is inserted;
5. The screws are tightened with a torque wrench.

After completing the above steps, the silicone is cured at room temperature for at least 2 h before the domes are released from the mold, and then it is post-cured for two more hours at 180°C. The purpose of the thermal curing is to ensure an optimal vulcanization of the silicone, which reduces the hysteresis in its response.

### 3.4 Dome Characterization

The characterization process aims at evaluating the dome force-displacement characteristic curve. To this end, an indenter connected to a linear motor (Aerotech, Inc., Model: ANT-25LA) is used to apply a 0.1 Hz sinusoidal displacement to the flat-top of the dome, while its reaction force is measured with a load cell (ME-Meßsysteme GmbH, KD40s) during both loading and the unloading paths. The amplitude of the displacement command is adapted to each individual dome geometry, and is chosen so that the complete region of negative stiffness is covered by the measurement. Due to the dissipative nature of the silicone, a hysteretic curve is observed. Such a behavior is mainly due to a structural (rather than viscous) damping processes occurring in the polymer, which is known to cause a rate-independent hysteretic response (Bishop, 1955; Ewins, 2009). This fact is confirmed by repeating the experiment at slower rates, which results in the hysteresis shape remaining unchanged (details are omitted for conciseness). Note that the performance of a DEA is negatively affected by any source of damping and hysteresis (caused, e.g., by the DE itself or the biasing element). For the considered types of DEA systems, damping mostly affects the stroke and the energy consumption.

The biggest influences on the manufacturing accuracy of the dome are expected to arise from the 3D printing process of the mold, as well as from the manual mixing of the silicone. To account for such inaccuracies, a total of eight batches are manufactured from three different molds having the same geometry. In this way, the effect of manufacturing tolerances on the reproducibility of the dome force-displacement curves can be properly quantified via repeated experiments.

**FIGURE 6:**
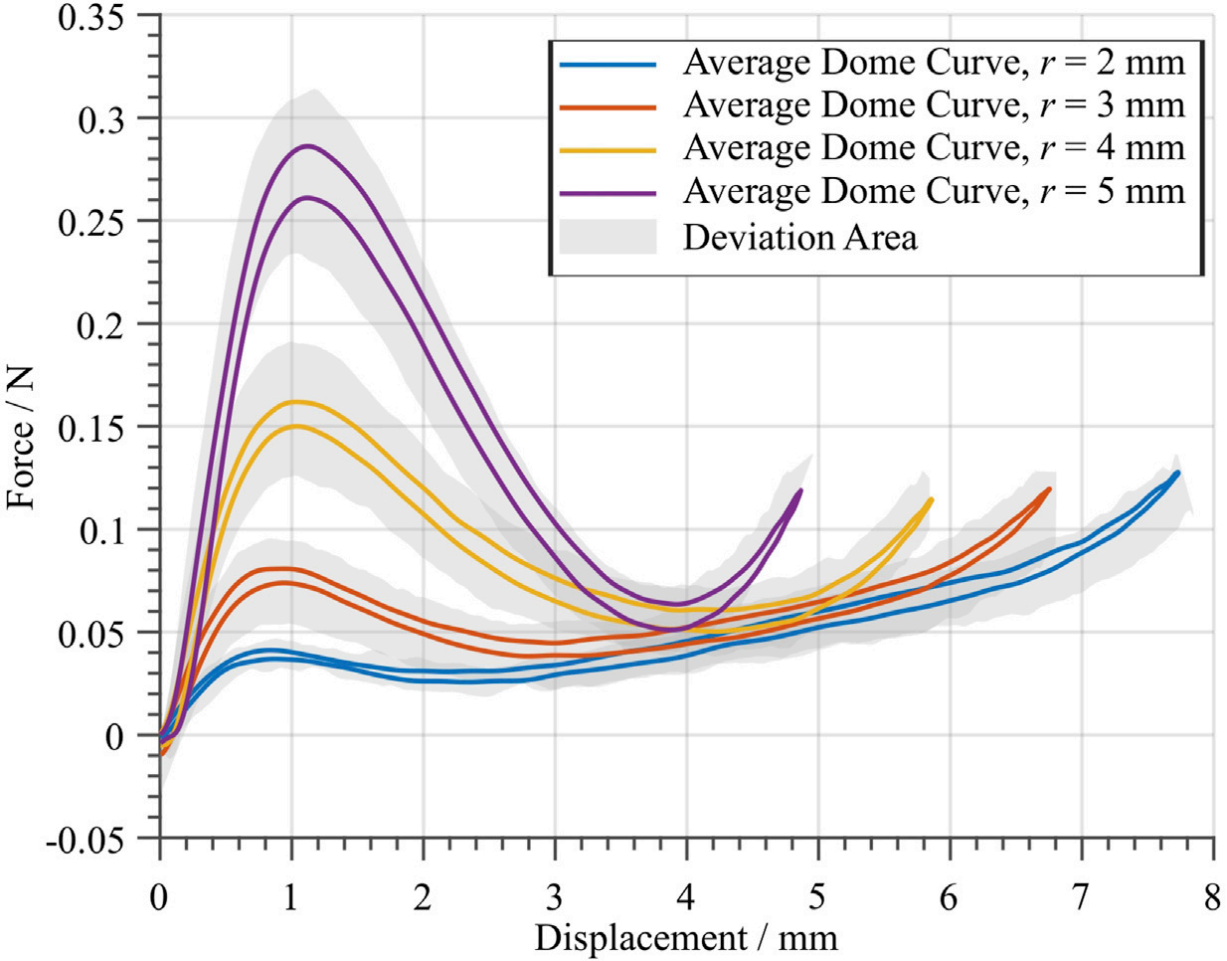
Average force-displacement curves of four different dome geometries. A total of eight batches were manufactured with three different molds, having the same geometry. Each solid curve is computed based on the average behavior between eight specimen, which all share the same nominal geometry. The gray area shows the maximum and minimum deviation of the measured specimen.

Figure 6 shows the results of the characterization of four different dome geometries. All of them share the same values of H=4 mm and t=600 μm, while *r* is chosen to four different values ranging from 2 to 5mm in steps of 1mm. These specific values of *H* and *t*, chosen on the basis of a preliminary investigations, lead to force-displacement curves having a desirable shape for the considered values of *r*. For each geometry, the solid line corresponds to the average between the eight measured specimens, while the grey region represents a range of maximum deviation among specimens. The results clearly shows the impact of *r* on the general shape of the curve. In particular, the value of *r* has a large impact on the force of the first local maximum, as well as on the value of the negative slope between the two local extrema. Similar families of curves can be also shown for other parameters. However, since they turned out not to be relevant for our specific dome design, which is tailored to a given COP-DEA geometry, they are omitted for conciseness. The observed deviations among the considered domes, which are supposed to share the same nominal geometry, reveal that higher values of *r* have a detrimental effect on reproducibility. However, the characterization shows that the value of the important design parameter $k_{\text{NBS}}$ is less affected in comparison to other curve features, i.e., the *F* value of the local maximum.

## 4 Dome Design Optimization

In this section, an application-specific dome design procedure is discussed. First, a target DE geometry is chosen, and its force-displacement curves are characterized at different voltage values. Then, a method is presented to optimize the free parameters of the dome, in such a way to match the given DE curves. In this way, a large stroke dome-DEA system can be effectively achieved.

### 4.1 Dielectric Elastomer Characterization

The first steps toward the development of a large-stroke soft DEA consist in the selection and characterization of a target DE geometry. As mentioned in Section 2.2, COP-DEAs with sputtered thin metallic electrodes (thickness of 10 nm) are chosen in this work. The material used for the elastomer membrane is a Wacker ELASTOSIL® film 2030, with an initial thickness of 50 μm. No significant amount of pre-stretch is applied during the manufacturing process. Outer and inner radii of these COP-DEAs are equal $R_{DE}=10\;\text{mm}$ and $r_{DE}=2.5\;\text{mm}$, respectively (cf. Figure 2A). Details on the manufacturing process can be found in Hubertus et al. (2020). In the planned assembly, the DE must be glued to the bottom of the dome base, whose radius has the same value of the inner radius of the dome (R=7.5 mm), see Figure 5A. Note that the outer electrode area in excess, which is glued to the base of the dome, does not undergo a stretching during actuation. Therefore, the value of *R* defines the overall size for the DE membrane. A double-sided silicone tape (Nitto P-905) is used for all the adhesive connections. Glues and adhesive tapes are preferred over screws or plastic frames, in order to achieve an overall flexible assembly, which is completely free of rigid parts.

The starting point for the actuator design consists of the evaluation of the DE characterization curves. To perform such a characterization, the sputtered DE is glued to a silicone ring having the same inner diameter R=7.5 mm as in the dome geometry. This ‘dome ring’ is manufactured in a similar fashion to Section 3, and fixes the outer DE radius as $R_{\text{DE}}=7.5\;\text{mm}$. Prior to gluing the DE to the dome ring, a thin piece of copper tape is used to implement the electrical contacts of the electrodes. The so-prepared DE is then ready to be characterized electro-mechanically. The characterization setup is the same one used for the dome, described in Section 3.4. To this end, a sine-wave out-of-plane displacement profile is applied to the center part of the membrane via the linear motor, while the reaction force is measured via the load cell. The amplitude and frequency of the displacement profile are selected equal to 7 mm and 0.1 Hz, respectively. The relatively slow displacement rate is chosen to minimize the viscoelastic material effects as best as possible. A further training cycle with an amplitude of 10 mm is performed before the actual characterization, to eliminate the Mullin’s effect. The radius of the indenter used for the displacement test determines the effective inner radius of the DE, thus it is chosen equal to the inner DE radius, i.e., $r_{\text{DE}}=2.5\;\text{mm}$. The experiment is performed two times, first without any external voltage, and then with a HV of 3 kV applied to electrodes, which is provided by a high-voltage amplifier (hivolt.de GmbH, HA51U series). In this way, the two characterization curves of the DE are obtained.

**FIGURE 7:**
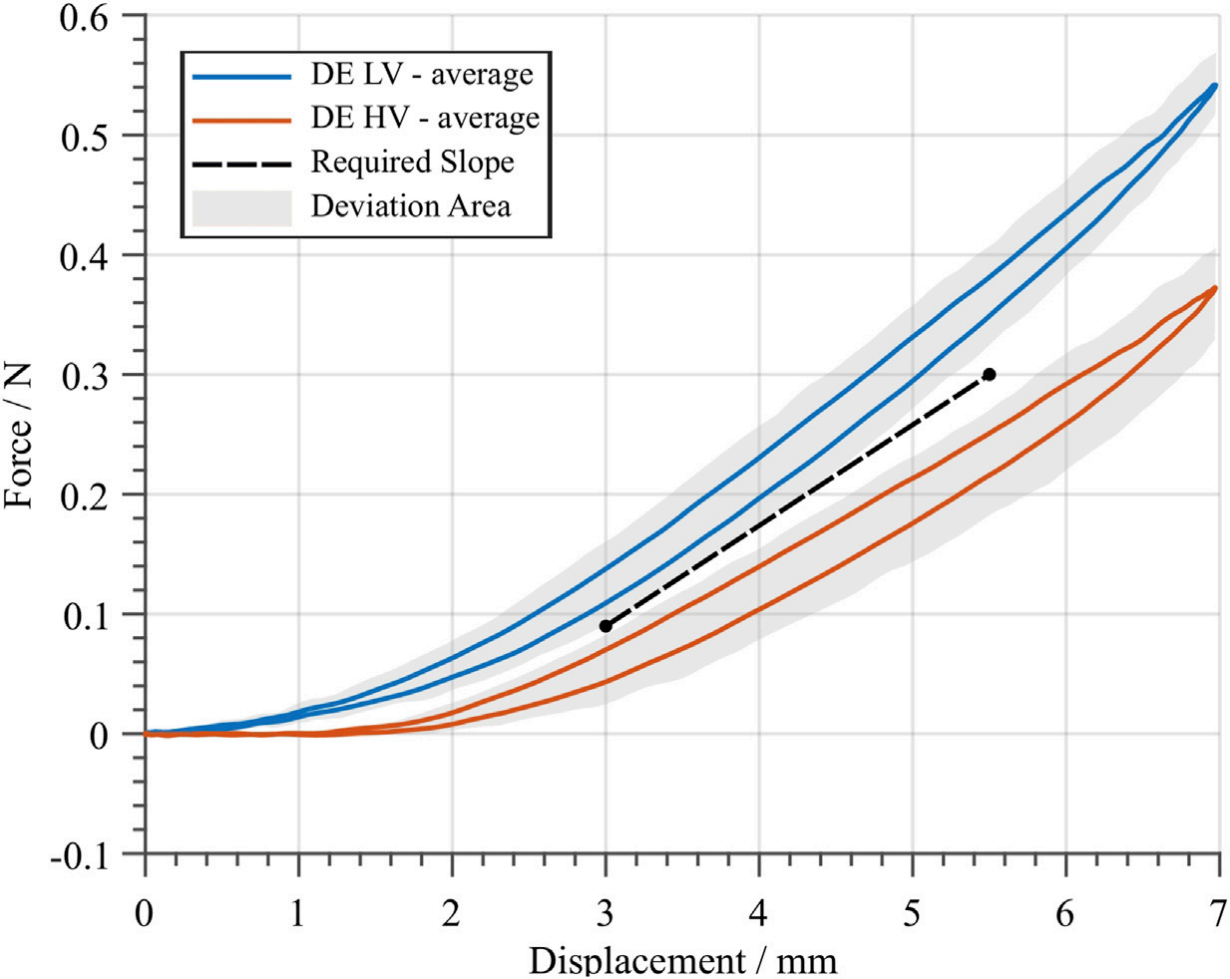
Average DE curves for the low- (blue) and high- (red) voltage case, based on the average between four different specimens. A sine-wave displacement profile with an amplitude of 7 mm and a frequency of 0.1 Hz was applied during the experiments. The gray area denotes the deviation among the four measured samples. The black dashed line represents the required stiffness $k_{\text{req}}$ of an ideal biasing system, which would lead to a large stroke performance. For the average DE curves, the slope of this line equals $k_{\text{req}}=0.084\;\text{N}/\text{mm}$.

The characterization experiment described above is repeated multiple times, by considering 4 DE membrane elements, in such a way to generate average curves for both high- and low-voltage cases. The results of the characterization process are shown in Figure 7. A residual hysteresis is observed, which is due to the intrinsically irreversible nature of the DE. The gray areas show the range of deviation among all the experiments, which depend on both manufacturing and assembling tolerances as well as on the repeatability of the measurement. These average curves can then be used to determine the ideal characteristics of an optimal biasing system, which ideally lies in the middle of the gap between the two DE curves. The characteristic of an ideal biasing system is reported as a dashed black line in Figure 7 (see Section 2.2 for details). Such a line is obtained by selecting two points corresponding to d=3 mm and d=5.5 mm, and result in a required NBS slope of $k_{\text{req}}=0.84\;\text{N}/\text{mm}$.

### 4.2 Dome Design Optimization

**FIGURE 8:**
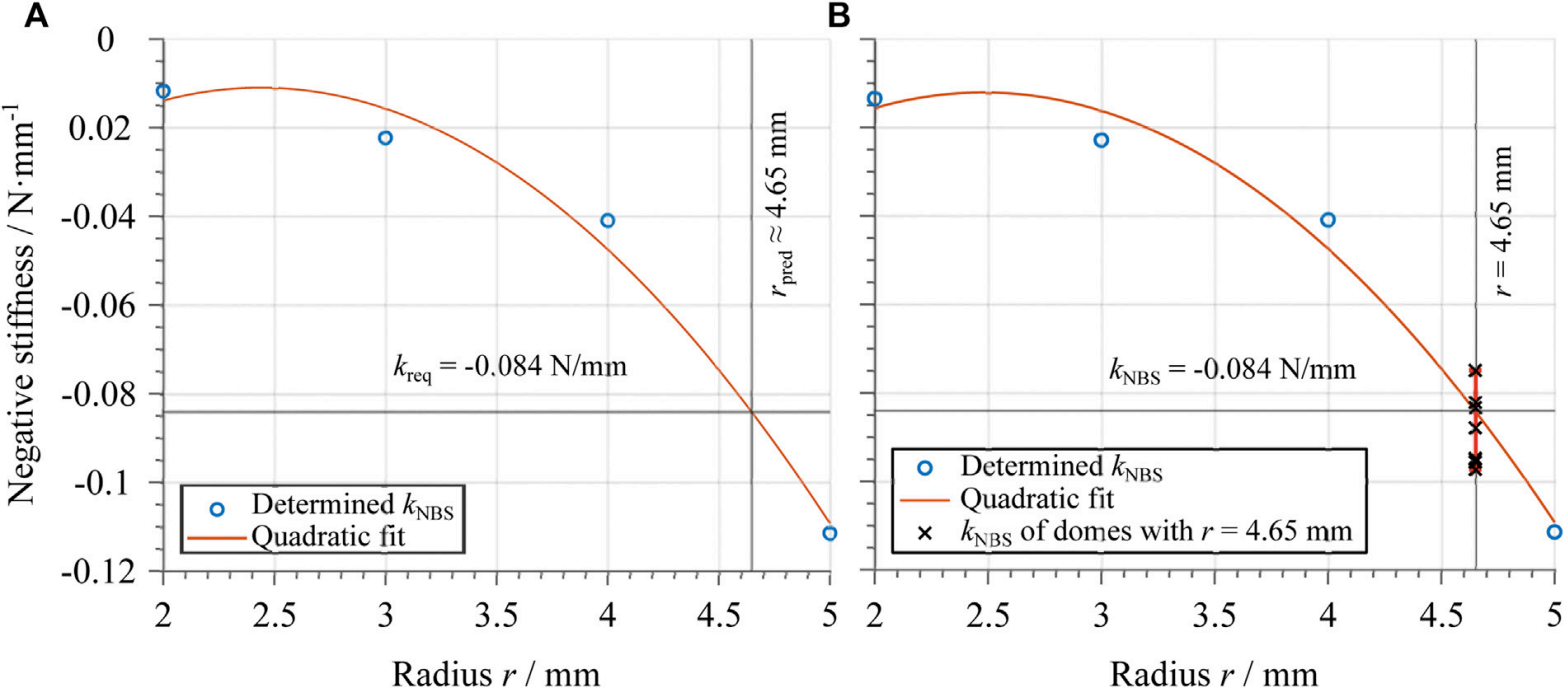
**(A)** Dependency of the negative stiffness of the dome average curves on the flat-top radius. The circles represent the determined values of the negative slope $k_{\text{NBS}}$, which are plotted against the corresponding value of the flat-top radius *r*. The values are extracted from the graphs shown from Figure 6, and are used to determine a quadratic fit (red line). The value of the predicted flat-top radius $r_{\text{pred}}$ is calculated based on this fit, by considering the value of the required negative slope $k_{\text{req}}$ reported in Figure 7. **(B)** Dependency of the negative stiffness of the dome average curves on the flat-top radius as in **(A)** together with the value of $k_{\text{NBS}}$ of eight individual optimal domes. The values of $k_{\text{NBS}}$ are shown with an errorbar, that states the deviation of the determined $k_{\text{NBS}}$ values.

In order to maximize the DEA system stroke, one must find an optimal dome geometry (in terms of *r*, *t*, and *H*) which matches as close as possible the required slope depicted in Figure 7. To perform an effective design, it is important to find a systematic approach which permits to shape the dome characteristic curves in a desired way. From Figure 6, it is evident that *r* has a strong influence on the negative slope region of the dome curve. We define $k_{\text{NBS}}$ as the slope of the dome curve computed at the center point of the negative stiffness region. The so-computed values of $k_{\text{NBS}}$ can then be plotted as a function of *r*, thus leading to the nonlinear trend shown in Figure 8A. By fitting the relationship between $k_{\text{NBS}}$ and *r* with a quadratic function, we can predict a certain value of $r_{\text{pred}}$ which corresponds to a target stiffness. Please note that the use of a quadratic function for the relationship between $k_{NBS}$ and *r* is just an empirical fit, rather than an accurate model of the structure. In reality, due to the kinematic complexity of the dome, the real relationship among those parameters is much more complex to describe. Nevertheless, since the obtained relationship mainly serves the purpose of deriving simple design rules for the dome, it is sufficient for the scope of this paper.

The approach described above is then applied by considering the required slope value $k_{\text{req}}=0.84\;\text{N}/\text{mm}$, which was previously computed in Section 4.1. The resulting optimal value of $r_{\text{pred}}$ is then estimated as $r_{\text{pred}}\approx 4.65\;\text{mm}$, as also reported in Figure 8A. While *r* is optimized based on the above described procedure, the values of *H* and *t* are left unchanged from the ones reported in Figure 6, for simplicity.

### 4.3 Optimal Design Validation

To validate the design in the previous section, various domes with the same optimal geometry (H=4 mm, t=600 μm, and r=4.65 mm) are manufactured and characterized in terms of negative slope. Figure 8B shows the same plot as in Figure 8A, together with eight negative slope values from the several optimal domes in the errorbar. It can be observed that the negative slope of the desired dome geometry lies within the expected range. Small deviations arise due to the tolerances in the manufacturing process, as well as to the quadratic dependency assumption for the $k_{\text{NBS}}-r$ relationship. Despite those small differences between theoretical and measured values of $k_{\text{NBS}}$, the computed geometry still allows the design of a functioning DEA. However, due to the possible variations in $k_{\text{NBS}}$, a preliminary evaluation phase is required to determine if the achieved dome characteristics is suitable for the given DE. This is explained in detail in the following section.

## 5 Actuator Manufacturing and Characterization

In this section, manufacturing and assembly of the overall DEA are first discussed. Then, an experimental characterization campaign is conducted to evaluate the full system performance.

### 5.1 Actuator Assembly and Manufacturing

**FIGURE 9:**
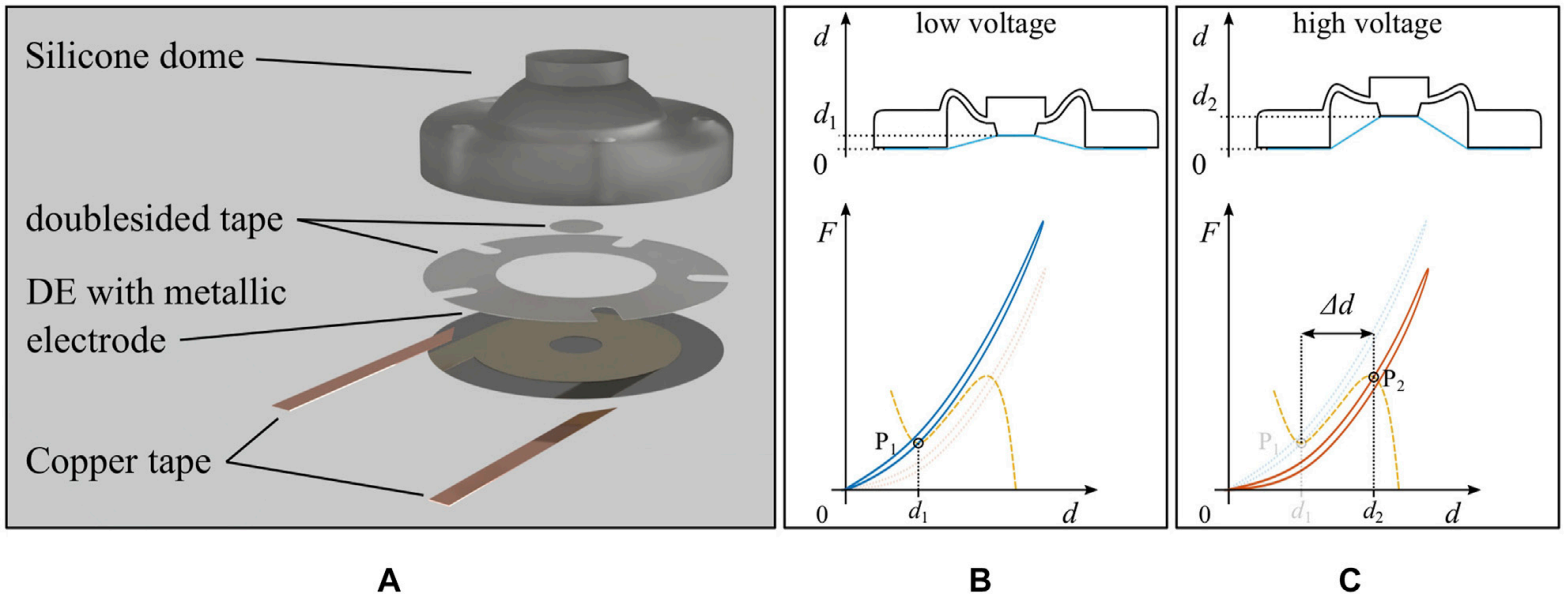
**(A)** Rendered explosion view of the flexible DEA. Sketch of the assembled actuator together with the system force-displacement curves, **(B)** without voltage and **(C)** with high voltage applied to the electrodes.

A rendered view of the whole assembly is shown in Figure 9A. The electrical connections with the metallic DE electrodes are achieved via copper tape, which is attached prior to the actuator assembly. Double-sided tape is cut with a cutting machine, and used for the connections between the center of the DE and the extension of the dome flat-top, as well as for the connection between the dome base and the outer DE part. Note how, in contrast to the very first dome-based DEA prototype presented in Neu et al. (2020), the mechanism in Figure 9A is designed to allow safer operations, since the high voltage controlled DE is placed underneath the isolating dome. Figures 9B,C further show the actuation principle of the considered actuator, by also illustrating the relationship between stroke Δd and force-displacement curves.

To allow for a more precise assembly in terms of adjustment between dome, tape, and DE, as well as a planar connection, several 3D printed tools are used. In this way a manual placement can be avoided, and thus a more accurate assembly can be achieved. Small channels at the bottom of the dome base ensure air ventilation, in order to prevent pressure changes in the volume between DE and dome during the actuation.

**FIGURE 10:**
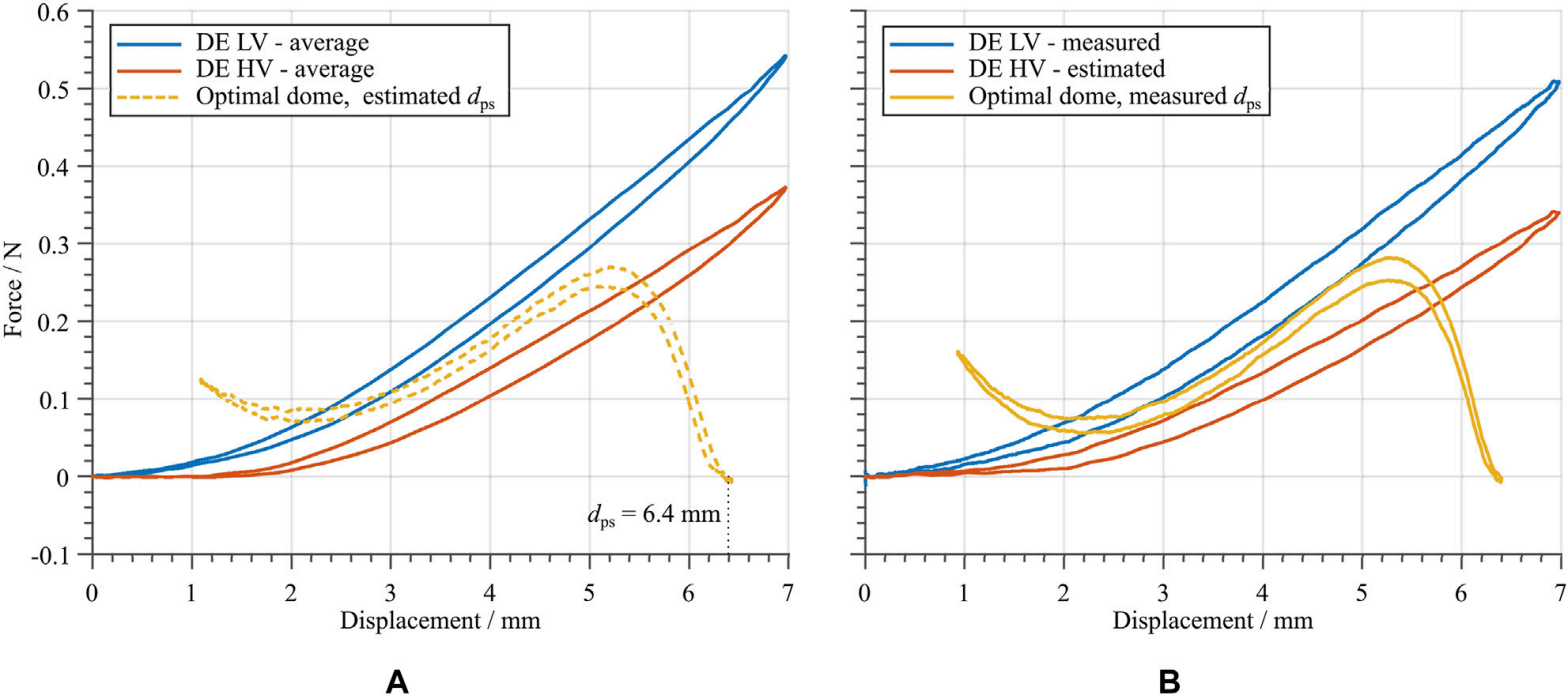
**(A)** Measured force-displacement curve of the dome, whose geometry is determined based on the optimization method in Section 4, together with the average DE curves from Figure 7, in order to check the suitability of the design and to determine the amount of pre-stretch. **(B)** Measured DE LV curve and estimated DE HV curve, together with a manufactured dome with the optimized geometry and value of $d_{\text{ps}}$ determined by a laser measurement.

The procedure needed to manufacture the system in Figure 9A is reported in the following, where the results of the most relevant steps are illustrated in Figure 10:

1. A suitable dome design is determined based on the optimization method explained in Section 4, and subsequently manufactured and validated;
2. By means of post-processing implemented in MATLAB, the optimal value of the horizontal shift between dome and DE curves, denoted as $d_{\text{ps}}$, is estimated. The resulting design is reported in Figure 10A;
3. Domes with different values of $d_{\text{ps}}$ are manufactured and characterized, by also including a laser measurement of $d_{\text{ps}}$ (Keyence, Model: LK-G87);
4. The DE which is used for the actuator is characterized prior to the assembly. Note that only the LV force-displacement curve is measured in this case, because it is not possible to nondestructively measure the DE HV curve, as long as the DE is not glued to the dome. This is done to improve the accuracy, and allows to determine if the curves of the used DE and the manufactured domes match properly;
5. By considering several manufactured domes, all sharing the same optimal design but differing in terms of $d_{\text{ps}}$, use MATLAB to test which dome fits the DE LV curve measured in the previous step as best as possible. In MATLAB, the measured dome curve is plotted together with the measured DE LV curve, by using the value of $d_{\text{ps}}$ determined via the laser measurement from step 3. The use of the measured value of $d_{\text{ps}}$, instead of the theoretical value, permits to increase the reliability of the design;
6. The overall actuator is assembled according to the method describe above in this Section.

The estimation of an appropriate range for $d_{\text{ps}}$ in step 2 is done via a graphical approach, based on Figure 3C. In step 3, domes with slightly different values of $d_{\text{ps}}$ are fabricated, in order to accommodate tolerances in the manufacturing process. Regarding step 4, it is remarked that even though the DE is only measured for the LV case, the HV characterization curve can be estimated by using the same gap obtained from the average DE curves reported in Figure 7. In this step, the DE is only measured without a voltage, due to practical difficulties encountered when applying a voltage prior to the assembly. When solely using the average DE curves from Figure 7 the reliability is negatively affected, due to the unavoidable manufacturing tolerances. Thus, the measurement of the DE LV curve allows to increase the accuracy of the overall process. In this way, it is still possible to use both DE curves to find the most suitable dome in step 5. In this case, however, the high-voltage curve is an estimated, rather than a measured one. The theoretically achievable maximum stroke with the shown dome curve and the average is approximately 2.8 mm.

### 5.2 Actuator Characterization

In order to characterize the manufactured DEA the stroke and energy consumption is measured for different voltage input signals and in several mechanically deformed configurations. To this end, a laser sensor (Keyence, Model: LK-G87) is used to measure the stroke of the flat-top of the DEA while the voltage signal is applied and an NI LabVIEW module (PXI-7852) is used to monitor an analog voltage output of the voltage amplifier, which corresponds to the current needed to charge and discharge the DE. To assess the validity of the design, the plot generated in step 5 of the manufacturing process in Section 5.1 provides the maximum expected stroke, which is approximately $d_{\text{max}}=2.8\;\text{mm}$.

**FIGURE 11:**
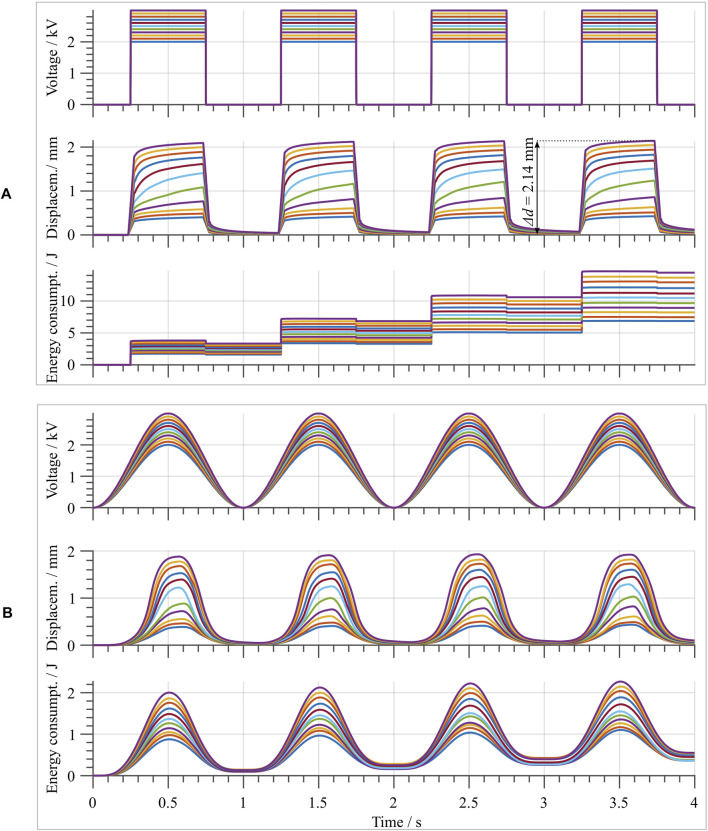
**(A)** upper part, shows the square wave voltage input signals with different amplitudes ranging from 2 to 3 kV in steps of 0.1 kV. Center and lower parts show the laser-measured stroke of the flat top, and the input energy supplied to the DEA for charging and discharging, respectively. **(B)** shows the same for sinusoidal voltage input signals with the same amplitudes as in **(A)**.

**FIGURE 12:**
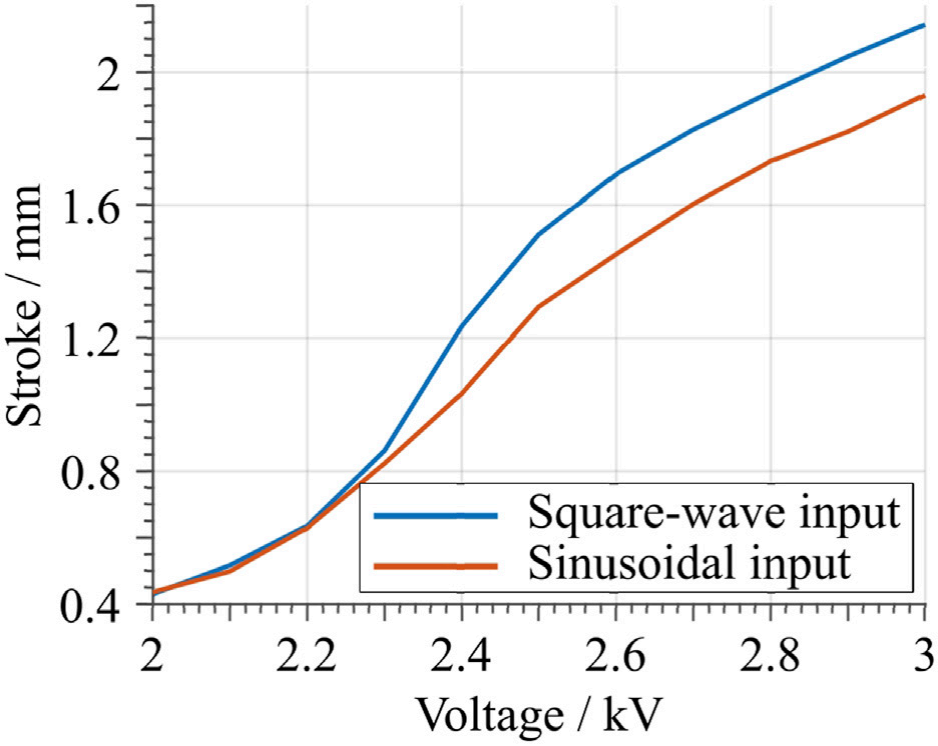
Maximum stroke as a function of the applied voltage amplitude. The blue curve represents the values obtained from the square wave input signal, while the red curve is obtained from the sinusoidal input.

To properly characterize the DEA performance, different excitation voltage signals are applied to the electrodes. In order to examine the dependency of the actuator stroke on the applied voltage amplitude, a square wave voltage signal is used as first test signal for the DEA. Square wave inputs with a frequency of 1 Hz, a duty cycle of 50%, and varying voltage amplitudes are considered in this phase. During actuation, the displacement of the flat-top is measured with the same laser sensor mentioned above. The tested voltage amplitudes range from 2 to 3 kV, with increments of 0.1 kV. The resulting curves are presented in Figure 11A. From this figure, the effects of the DE material creep can readily be observed. The highest achieved stroke in this measurement is approximately 2.14 mm, which is less than the maximum theoretical stroke of 2.8 mm. This might be due to tolerances of the assembly, as well as to measurement tolerances occurring during the manufacturing process (cf. Section 5.1). Figure 12 shows the maximum stroke as a function of the amplitude of the applied voltage. Such a plot also highlights the controllability feature of the developed actuator. Finally, the input energy performance are reported in the last row of Figure 11A. The energy is estimated by means of the current monitor of the HV amplifier. From here, it can be noted how the actuator absorbs energy when actuated, and releases electric energy when deactivated. The ratio between released and absorbed energy allows us to estimate an energy efficiency figure of about 9.63%, by considering the average value among all the tested voltages. This value appears as low due to the particular choice of the input signal (i.e., a step), which is known to be unsuitable for energy-optimal activation of capacitors.

The same characterization is then repeated by considering a sinusoidal voltage input, by using the same voltage amplitudes as for the square wave case. The obtained results are shown in Figure 11B. When the maximum measured stroke is compared to the one obtained with the square wave input, given the same voltage value, a slight decrease is observed. This becomes evident in Figure 12, when the blue (square-wave) and red (sine-wave) curves are compared. The decrease can be explained by the creep due to the hysteresis of the material system, which is also observed with the square-wave input. At the same time, the total energy consumption is drastically smaller for the sine-wave input. This is due to the lower frequency content of the input signal, which results in overall smaller dissipation. For this case, an average energy efficiency figure of 90.96% is estimated. Note how this value is significantly higher compared to the previous square wave case.

**FIGURE 13:**
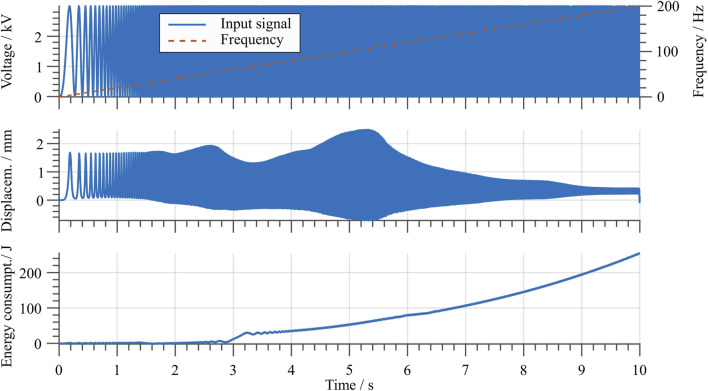
Stroke and energy consumption with a sine sweep input signal. The top graph shows the input signal in blue and the frequency over time with a dashed red line.

Additionally, to determine the actuator dynamic range of operation, a sine-sweep input voltage test is conducted. The considered signal has a constant amplitude of 3 kV, while its frequency ranges from 1 to 200 Hz with a linear increment of 20 Hz/s. The resulting actuator stroke is measured with the laser sensor, and is shown in Figure 13. In here, it can be clearly observed how the resonance frequency of the first mode is located at around 106 Hz, and that for further increasing driving speeds the stroke is significantly decreasing. This can be explained by considering the hysteretic behavior of the dome as well as of the DE itself. In fact, the viscoelasticity of such polymeric components results into a damping effect which, in turn, causes the output stroke to decrease. Note also that, once a certain frequency of approximately 60 Hz is exceeded, a drastic increase of energy consumption is observed. This is due to the natural high-pass characteristics of the DE, which results from the combined effects of electrical and mechanical (i.e., viscoelastic) losses occurring in the system.

**FIGURE 14:**
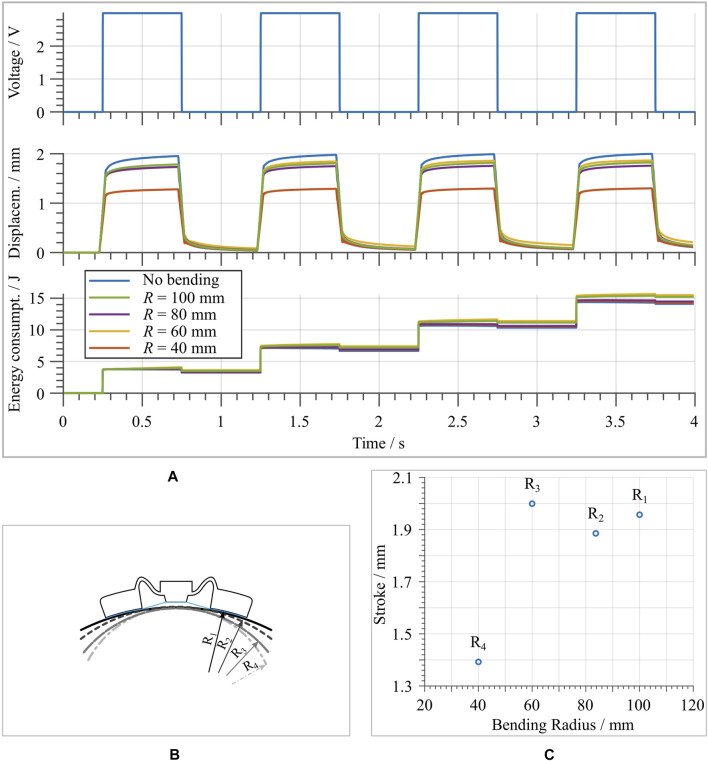
**(A)** Stroke and energy consumption measurement of the actuator in different deformed configurations, together with the results in the undeformed state as reference. **(B)** Cross-sectional schematic of the applied deformation. Note that the deformation corresponds to a cylindrical, rather than spherical, shape. **(C)** shows the maximum stroke as a function of the curvature radius.

In Section 1 the idea of using an array of multiple flexible DEAs as a smart, deformable skin was introduced. In order to show the suitability of the developed soft DEA for such kinds of applications, the actuator was also characterized when subject to a state of mechanical deformation. In particular, the DEA is characterized while being deformed according to different curvature values, as if it is attached to various cylinders with different radii. For a first evaluation, four different curvature radii are chosen, i.e., 100, 80, 60 and 40 mm, see Figure 14B. The results are shown in Figure 14A, in terms of both stroke and energy consumption. The amplitude of the square wave excitation signal is 3 kV, for all of the shown curves. To provide a reference, the results of the undeformed actuator is also reported in the plot. It can be seen that the deformed DEA is still able to operate in each one of the four configurations, even thought it is clearly losing actuation performance for the smallest tested bending radius of 40 mm. When only the maximum stroke is considered as a function of the bending radius, the graph in Figure 14C is obtained. Despite the collected data are not enough to extrapolate a law between bending radius and stroke, the plot effectively showcases the ability of the actuator to perform while being heavily deformed.

## 6 Conclusion

In this work, a novel concept for soft, flexible, and large-stroke DE actuators was presented. The system features a polymer-based biasing element, namely a three dimensional silicone dome, manufactured via a casting process. The polymeric dome serves as a negative-rate bias spring element, which allows to magnify the output stroke of a circular DEA while adding an overall flexibility and deformability to the whole system. Therefore, it appears as a suitable solution for manufacturing flexible and high-stroke DEAs, capable of meeting the compliance requirements of soft robotic applications.

After discussing the dome manufacturing principle, a design optimization was performed with the aim of finding the dome geometry which maximizes the DEA stroke. Performance characterization of the novel dome-based DEA system were then conducted in terms of stroke output and energy consumption, for both static and dynamic operating regimes. The results of this characterization revealed an out-of-plane maximum stroke of about 2.1 mm for an outer actuator radius of 7.5 mm, and energy efficiency higher than 90% in case sufficiently smooth control signals are applied. To showcase the suitability of the dome-DEA for soft robotics and wearables, a characterization was also conducted while keeping the complete actuator in a deformed state. The DEA was still capable of operating with an unchanged stroke performance when deformed with a curvature radius larger or equal than 60 mm, while its stroke dropped considerably in case of curvature radius equal to 40 mm.

To conclude, we point out that the design optimization performed was based on solely changing one design parameter of the dome layout. In future research, we will study the effects of changing all of the free design parameters via model-based approaches, to allow a further optimization of the DEA stroke. Miniaturization of the considered actuator will also be studied, with the aim of developing micro-arrays of cooperative DEAs.

## Data Availability

The original contributions presented in the study are included in the article/Supplementary Material, further inquiries can be directed to the corresponding author.
